# Mortality associated with compound exposure to heat and ozone across five Moroccan regions

**DOI:** 10.21203/rs.3.rs-10671369/v1

**Published:** 2026-10-12

**Authors:** Mouaad Adarbaz, Kenza Khomsi, Imad Ilbadisy, Annas Houdou, Jamila El Mendili, Abdelilah El Marnissi, Mohamed Khalis

## Abstract

**Background** : Compound heat-ozone events are projected to become more frequent and intense under climate change, with North Africa among the most exposed regions, yet evidence from this region remains scarce. **Methods** : To quantify pooled and region-specific excess mortality associated with heat, ozone, and their co-occurrence across five Moroccan regions in 2021. We used a time-stratified case-crossover analysis of daily all-cause mortality using conditional quasi-Poisson models. Extreme heat and ozone were defined as days with maximum temperature (Tmax) and maximum daily 8-h average ozone (MDA8) ≥90 ^th^ of region-specific percentile, and compound heat-ozone events as same-day co-exceedance or higher ozone within the subsequent. **Results:** In the pooled analysis, heat-only and compound events were associated with statistically significant excess mortality (RR=1.18, 95% CI:1.11-1.1.25 and RR=1.16, 95% CI:1.10-1.22 respectively), ozone-only days showed no statistically significant excess mortality. The region-specific analysis showed a substantial heterogeneity, with Oriental was the only region with significant associations across all exposures. A significant heat-only association was also found in Tanger-Tétouan-Al Hoceima (RR=1.22, 95% CI: 1.11-1.35). The analysis of interaction metrics showed no sign of synergistic interaction between heat and ozone. Results were stable across different lag specifications and sensitivity analysis excluding extreme COVID-19 periods and individual regions. **Conclusion:** extreme heat alone or compounded with high ozone was associated with excess mortality in Morocco. This risk was spatial heterogeneous and not amplified by ozone. These results support region-specific early warning system and stress the need for longer time series to refine compound exposures risk estimates

